# Transcriptional factors associated with epithelial-mesenchymal transition in choroidal neovascularization

**Published:** 2011-05-06

**Authors:** Manabu Hirasawa, Kousuke Noda, Setsuko Noda, Misa Suzuki, Yoko Ozawa, Kei Shinoda, Makoto Inoue, Yoko Ogawa, Kazuo Tsubota, Susumu Ishida

**Affiliations:** 1 Laboratory of Retinal Cell Biology, Keio University School of Medicine, Tokyo, Japan; 2 Department of Ophthalmology, Keio University School of Medicine, Keio University School of Medicine, Tokyo, Japan; 3 Department of Ophthalmology, Hokkaido University Graduate School of Medicine, Sapporo, Japan; 4 Department of Nursing, Tokai University School of Health Sciences, Isehara, Japan; 5 Department of Ophthalmology and Visual Science, Yokohama City University Graduate School of Medicine, Yokohama, Japan; 6 Department of Ophthalmology, Teikyo University School of Medicine, Tokyo, Japan; 7 Kyorin Eye Center, Kyorin University School of Medicine, Tokyo, Japan

## Abstract

**Purpose:**

To investigate the transcriptional factors associated with epithelial-mesenchymal transition (EMT) in choroidal neovascularization (CNV) secondary to age-related macular degeneration (AMD).

**Methods:**

Paraffin sections of CNV obtained from patients with AMD (n=12) were stained for transcriptional factors related to EMT, *i.e.*, Snail, Slug, SIP1, and Twist. As a control, postmortem sections of ocular normal tissue were used. Furthermore, using a human retinal pigment epithelial (RPE) cell line (ARPE-19), reverse transcription–polymerase chain reaction (RT–PCR) and immunofluorescence microscopy were performed to explore the cellular localization and expression levels of EMT-associated transcriptional factors upon cytokine stimulation.

**Results:**

Of 12 specimens, 11 CNV tissues (91.6%) showed staining for Snail localized in cellular nuclei, particularly in those of RPE cells. Snail was strongly co-localized with α-smooth muscle antigen (SMA) in RPE cells. In contrast, postmortem human retina showed no Snail staining in RPE cells. Other transcriptional factors, Slug, Twist and SIP1 were not detected in CNV or normal human retina. In ARPE-19 cells, RT–PCR and immunofluorescence microscopy showed that Snail mRNA was upregulated by transforming growth factor (TGF)-β and VEGF stimulation. Furthermore, TGF-β induced relocalization of Snail to the nucleus in RPE cells.

**Conclusions:**

The current data indicate that Snail is a major transcriptional factor for EMT changes of RPE cells in human CNV.

## Introduction

Wet age-related macular degeneration (AMD), characterized by the formation of choroidal neovascularization (CNV), is a leading cause of irreversible blindness among people over age 50 in the western world [1]. So far, the mechanisms of CNV formation have been well analyzed, and lines of evidence have revealed that angiogenesis, inflammation, and oxidative stress are the underlying causes [1-7]. The accumulated data have led to the development of several therapeutic strategies for AMD, such as verteporfin photodynamic therapy (PDT) [8], anti-vascular endothelial growth factor (VEGF) therapy [9], and combined therapy [10,11]. By contrast, little is known regarding the molecular mechanism(s) of tissue scar formation in CNV. Since fibrotic changes in the foveal CNV lesion result in permanent visual impairment in patients with wet AMD [12], the mechanisms of tissue fibrosis in the late stage of AMD are of great interest.

Epithelial-mesenchymal transition (EMT) plays a role in physiologic and pathological conditions, for instance, in embryogenesis, tumor progression, invasion, metastasis, and tissue fibrosis [13]. During EMT, epithelial cells lose their characteristics and acquire the properties of mesenchymal cells. Upon tissue repair-associated events and tissue fibrosis, EMT is known to be triggered by inflammatory cytokines, cytotoxic stress, and DNA damage [13]. Similarly, upon CNV formation retinal pigment epithelial (RPE) cells have been shown to lose their junctional integrity [14] and convert morphologically to fibroblastic-shaped cells [15,16]. These facts led us to the hypothesis that EMT of RPE cells is involved in the fibrotic scar formation in AMD. Indeed, it has been demonstrated that EMT is crucial in the formation of matrix deposition and tissue fibrosis in ocular tissues [17,18]. Furthermore, inflammatory or angiogenesis-related cytokines, such as VEGF, connective tissue growth factor (CTGF), tumor-necrosis factor (TNF)-α, and transforming growth factor (TGF)−β, which are known to trigger EMT changes, are found in CNV tissues [5,15,19-21]. However, whether and to what extent EMT occurs during the tissue fibrosis of CNV tissue remains unclear.

An important group of transcriptional factors inducing EMT is the Snail family, including Snail (Snail1) and Slug (Snail2), zinc finger proteins that regulate changes in gene-expression patterns. The Snail family suppresses the expression of epithelial molecules like E-Cadherin and zonular occludens (ZO)-1 by binding to the DNA promoter region and stimulating mesenchymal changes, cell mobility, and proliferation in a variety of epithelial cells [22-24]. Furthermore, clinical studies have shown that upregulation of Snail family expression is observed in various tumors originating from epithelial cells [25-28] and in renal fibrosis [29,30], indicating the pivotal role of Snail during EMT. SIP1 (Smad interacting protein 1) is also a zinc finger protein, postulated as an invasion promoter suppressing epithelial adhesion molecule transcription [31]. Snail and SIP1 bind to overlapping promoter sequences and show similar silencing effects [32]. Another molecule known to trigger EMT mechanisms is Twist, a transcription factor containing a helix–loop–helix DNA-binding domain that regulates the Cadherin gene family [33].

The aim of this study is to explore the expression of EMT-associated transcriptional factor(s) in CNV and to determine whether cytokines found in human CNV tissues upregulate the transcription factor(s) for EMT in RPE cells.

## Methods

### Specimens

CNV tissues were collected from 12 eyes of 12 patients with AMD (1 eye per patient; 8 males and 4 females, mean age, 67.3±5.2 years old), who underwent pals plana vitrectomy to remove the subfoveal CNV at the Keio University Hospital from 2000 to 2001. The CNV tissues were fixed in 4% PFA immediately after the excision and embedded in paraffin. Serial sections were prepared on glass slides for immunohistochemical staining. Patient records were retrospectively reviewed for clinical characteristics, which are summarized in Table 1. No PDT or intravitreal injection of anti-VEGF agent had been administered in any of the cases before the surgical intervention. The current study was conducted in accordance with the provisions of the Declaration of Helsinki, and written informed consent was obtained before the surgery. The study protocol was approved by the Institutional Review Committee of the Keio University School of Medicine (Tokyo, Japan).

**Table 1:** Clinical Information.

**Number**	**Age**	**Gender**	**Eye**	**Visual acuity**	**Past ocular history**
1	64	M	L	20/250	
2	72	M	L	20/200	
3	55	F	R	20/2000	
4	69	M	R	20/100	
5	65	M	R	20/2000	CSC
6	71	M	L	20/300	
7	62	F	R	20/200	CSC
8	73	M	R	20/300	
9	73	F	L	20/50	
10	68	M	R	20/100	CSC
11	69	F	R	15cm/CF	
12	67	M	L	20/500	

CF, counting fingers; CSC, central serous chorioretinopathy.

As a control, two postmortem sections of ocular tissues with no retinal diseases (1 section per male from 60- and 62-year-old Caucasian males), obtained from Northwest Lions Eye Bank (Seattle, WA), were used. In addition, normal retinal tissues were obtained from 40-week-old C57Bl/6 mice (n=3; CLEA, Tokyo, Japan), after the animals were sacrificed with an overdose of anesthesia (Ketamine and Xylazine). The animal experiment was conducted in accordance with the ARVO Statement for the Use of Animals in Ophthalmic and Vision Research.

### Cell culture

The human RPE cell line ARPE-19 was cultured in a 1:1 mixture of Dulbecco’s modified Eagle’s medium and Nutrient Mixture F-12 (Invitrogen, Carlsbad, CA), supplemented with 10% (vol/vol) fetal bovine serum (Cambrex, Walkersville, MD), 50 U/ml penicillin, and 50 μg/ml streptomycin on a 35 mm or 60 mm dish (Falcon, Los Angeles, CA). Culture medium was replaced 3 times a week. Cells were maintained at 37 °C, 5% CO_2_ in a humidified atmosphere.

### Immunohistochemistry and morphometric analysis

Serial paraffin sections were de-paraffinised and rehydrated with a graded series of ethanol. The sections (n=3) were treated for antigen retrieval by pressure boiling in citrate buffer (0.01M, pH 6.0) for 4 min at 121 °C, washed with 0.1% Triton-X, and blocked with 10% normal goat serum (Dako, Carpinteria, CA). Subsequently, the sections were reacted with primary antibodies at 4 °C overnight: RPE65 (specific marker for RPE: Transduction Laboratories, Lexington, KY) at a dilution of 1:100, α-smooth muscle actin (SMA; Sigma, St. Louis, MO) at a dilution of 1:500, Snail (Abcam, Cambridge, MA) at a dilution of 1:100, Slug (Abcam) at a dilution of 1:250, Twist (Abcam) at a dilution of 1:650, and SIP1 (Abcam) at a dilution of 1:200. After washing, the sections were incubated with Alexa 488 or Alexa 546 conjugated goat anti-rabbit or mouse IgG (Invitrogen) diluted 1:200 in PBS for fluorescent signal detection. The nuclei of cells were counterstained with Hoechst 33258 (Sigma). All antibody concentrations were determined individually by appropriate positive controls. Primary antibodies of the same isotype (Santa Cruz, Santa Cruz, CA) were used as negative control. For morphometric analysis, serial sections (n=3) of CNV were stained by hematoxylin and eosin, and the degree of staining for EMT-associated transcriptional factors and tissue fibrosis was evaluated based on a 4 grade scale: - (negative), + (10% to 40%), ++ (40% to 70%), or +++ (70% to 100%).

ARPE-19 cells were cultured and then were treated with recombinant human TGF-β (10 ng/ml; R&D systems Minneapolis, MN) or VEGF (10 ng/ml; R&D systems) for 8 h. Cells were then fixed with cold methanol/acetone (1:1) for 5 min and permeabilized with 0.1% Triton X-100 for 20 min and were blocked with 10% normal goat serum (Dako, Carpinteria, CA) for 20 min. Cells were incubated with the following primary antibodies at 4 °C overnight: Snail (goat anti-rabbit; Abcam) and N-cadherin (BD Transduction Laboratories, Franklin Lakes, NJ). Cells were then treated with the Alexa 546-conjugated secondary antibody (goat-anti mouse or rabbit, 1:200; Invitrogen) for 60 min. The nuclei of cells were counterstained with Hoechst 33258 (Sigma).

### Reverse transcription PCR

ARPE-19 cells were cultured in serum free medium for 24 h and then exposed to TGF-β (10 ng/ml), TNF-α (10 ng/ml; Calbiochem, San Diego, CA), VEGF (10 ng/ml), CTGF (10 ng/ml; Cell Science, Canton, MA), bFGF (10 ng/ml; Invitrogen), and IGF-1 (10 ng/ml; R&D systems) for 4 h at 37 °C. Total RNA was extracted from ARPE-19 cells by the Trizol reagent (Invitrogen) and reverse transcription (RT) was performed using the First-Strand cDNA synthesis kit (GE Healthcare, Buckinghamshire, UK) according to the manufacturer's instructions. PCR products were obtained after 30 cycles of amplification with an annealing temperature of 55 °C using a platinum PCR supermix (Invitrogen). PCR primers were as follows: 5′-AAT CGG AAG CCT AAC TAC AG −3′ (forward), 5′- GGA AGA GGC TGA AGT AGA G −3′ (reverse) for Snail [34]; 5′- GAA GAG CTA CGA GCT GCC-3′ (forward), and 5′- TGA TCC ACA TCT GCT GGA-3′ (reverse) for β-actin [35]. PCR products were subjected to electrophoresis on 1.5% agarose gels and visualized by ethidium bromide staining. Quantification of band density was performed using NIH Image 1.41 software (developed by Wayne Rasband, National Institutes of Health, Bethesda, MD). The experiments were performed in duplicate and independently repeated 3 times.

### Statistical analyses

All results were expressed as mean±SEM with n-numbers as indicated. Student’s *t* test was used for statistical comparison between the groups. Differences were considered statistically significant at p<0.05.

## Results

### Transcriptional factors for EMT in human CNV tissues

**Figure 1:**
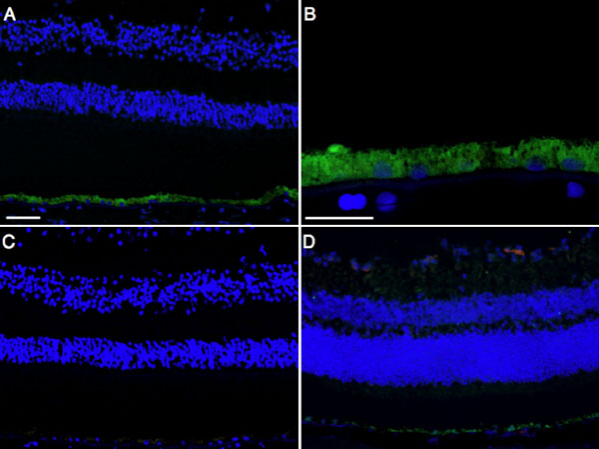
Immunolocalization of Snail protein in normal retina. **A**: Representative immunofluorescent micrograph of Snail (red), RPE65 (green), and nuclear counterstaining with Hoechst 33258 (blue) in normal human retina. **B**: High magnification. **C**: Negative control. **D**: Merged image of a representative immunofluorescent micrograph of Snail (red), RPE65 (green), and nuclear counterstaining with Hoechst 33258 (blue) in normal mouse retina. Scale bars for **A**, **C**, and **D** shown in **A**=50 µm, for **B**=25 µm.

To determine whether transcriptional factors for EMT are expressed in retinal tissue under normal conditions, we first stained normal human and mouse retina with antibodies against EMT-associated transcriptional factors. Snail was not detected in the RPE layer of normal retina (Figure 1). Similarly, the other transcriptional factors for EMT, Slug, Twist, and SIP1 were also undetectable in the RPE layer of normal retina (data not shown).

**Figure 2:**
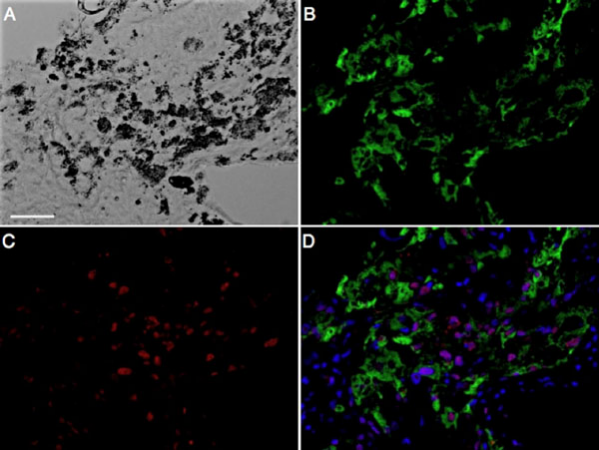
Immunolocalization of Snail protein in human CNV. Representative micrographs of human CNV section. **A**: Phase contrast image. **B**: Fluorescent micrograph of RPE65 (green). **C**: Fluorescent micrograph of Snail (red). **D**: Merged Image. Nuclei were counterstained with Hoechst 33258 (blue). Scale bar=50 µm.

Next we stained for these same factors in surgically excised CNV tissues, tissues composed of blood vessels, stromal cells, and pigmented cells in a fibrous interstitium. The pigmented cells are stained with RPE65, indicating that the cells originated from RPE cells (Figure 2A,B). In CNV tissues, Snail was predominantly detected in the nuclei of the pigmented cells (Figure 2C,D), but it was also found in the nuclei of fibroblast-shaped cells and vascular endothelial cells. Signals for Slug, Twist, and SIP1 were undetectable in the cell nuclei of the CNV tissues (data not shown).

**Figure 3:**
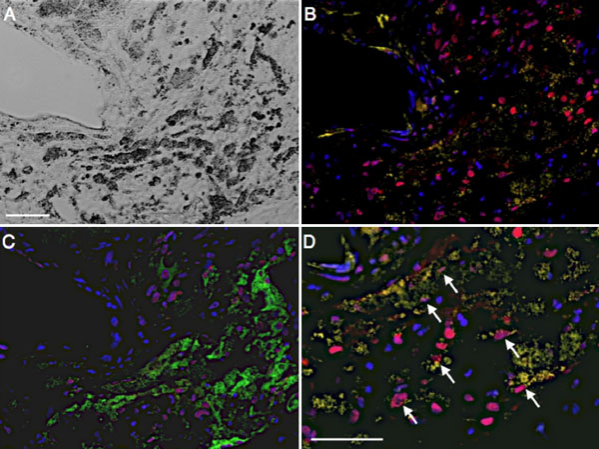
Co-localization of Snail protein and α-SMA in human CNV. Micrographs of a representative section of human CNV. **A**: Phase contrast image. **B**: Fluorescent micrograph of α-SMA (yellow), Snail (red), and cell nuclei (blue). **C**: Consecutive section stained for RPE65 (green), Snail (red), and cell nuclei (blue). **D**: High magnification of **B**. Arrows indicate the co-expression of α-SMA and Snail in RPE cells. Scale bar shown in **A** and **D**=50 µm.

Immunostaining for Snail in the nuclei of RPE65-positive cells was found in 11 of 12 CNV specimens (91.6%, Figure 2 and Table 2). Morphometric analysis showed that CNV tissues with Snail-positive RPE cells (++ or +++) are associated with higher fibrotic changes (++ or +++) in comparison with those containing less Snail-positive RPE cells (Table 2), indicating a relationship between Snail expression and tissue fibrosis in RPE cells. On the cellular level, Snail–positive RPE cells (++ or +++) co-expressed α-SMA (Figure 3, Table 2).

**Table 2:** Snail expression and tissue fibrosis.

**Number**	**Snail-positive RPE**	**SMA-positive cells**	**Tissue fibrosis**
1	+	+	++
2	++	+	++
3	+++	++	+++
4	+++	++	+++
5	+	++	+
6	-	-	+
7	+	-	+
8	++	+	++
9	++	+	+
10	+++	+++	+++
11	+	-	+
12	++	+	++

### Snail expression in cultured human RPE cells

**Figure 4:**
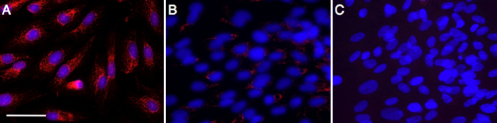
Cellular Localization of Snail in ARPE-19 cells. **A**: Cellular localization of Snail (red) at 2 days after passage. Snail is observed in both the nuclei and cytoplasm. Nuclei were counterstained with Hoechst 33258 (blue). **B**: Cellular localization of Snail (red) at 7 days after passage. Snail expression is decreased and observed mainly in the cytoplasm, but not in the nuclei. **C**: Negative control. Scale bar=50 µm.

To explore the cytokines that induce transcription of Snail in RPE cells, we stimulated ARPE-19 cells with candidate cytokines and examined the levels of *Snail* mRNA. First, to determine whether RPE cells constitutively express Snail, we stained ARPE-19 cells with the antibody against Snail. At 2 days after passage, Snail was strongly stained in both the cell nuclei and cytoplasm of cultured RPE cells (Figure 4A). By contrast, after cell-cell contact was established at 7 days after passage, the signal intensity for Snail was reduced in the nuclei and showed faint staining in the cytoplasm compared to those at 2 days after passage (Figure 4B), suggesting that production and cellular localization of Snail in RPE cells is related to the cell density and/or maturation of cell-cell contact.

**Figure 5:**
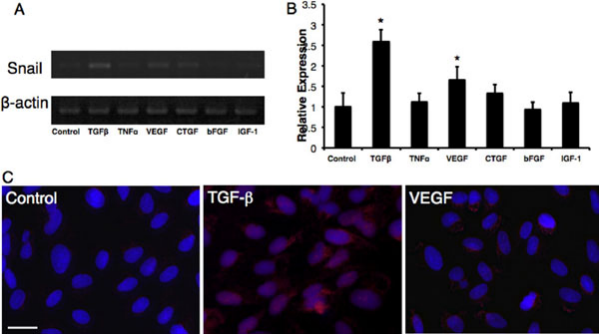
Snail expression after cytokine stimulation in ARPE-19 cells. **A**: RT–PCR amplification of Snail mRNAs from ARPE-19 cells after cytokine stimulation. ARPE-19 cells were incubated with PBS or recombinant human TGF-β, TNF-α, VEGF, CTGF, bFGF, and IGF-1. After 4 h, RNA was extracted from these cells. Snail mRNA expression was measured by reverse-transcription PCR. **B**: Gel densitometric analysis. Values are mean±SEM (n=6 in each group). *p<0.05. **C**: Immunofluorescence for Snail after cytokine stimulation. Left-Control. Middle-8 h after TGF-β stimulation (10 ng/ml). Right-8 h after VEGF stimulation (10 ng/ml). Scale bar=25 µm.

Next, at 7 days after passage we stimulated ARPE-19 cells with cytokines, TGF-β, TNF-α, VEGF, CTGF, bFGF, and IGF-1, previously found in human CNV samples. Among them, TGF-β and VEGF significantly upregulated *Snail* mRNA (Figure 5AB). However, the mRNA level of *Snail* was not changed with stimulation of the other cytokines. Furthermore, fluorescence microscopy depicted the enhanced staining of Snail in the nucleus and cytoplasm of ARPE-19 cells stimulated with TGF-β at 7 days after passage (Figure 5C), indicating a role for TGF-β in the upregulation and nuclear relocalization of Snail in RPE cells. By contrast, VEGF enhanced immunoreactivity of Snail mainly in the cytoplasm, but not in the nucleus (Figure 5C).

### Snail and N-cadherin in cultured human RPE cells

To study the relationship between Snail and adherence junctions in epithelial integrity, we stained ARPE-19 cells with antibodies against Snail and N-cadherin before and after establishment of cell-cell contact and with or without TGF-β stimulation. The major cadherin expressed by RPE cells in culture is N-cadherin [36], making N-cadherin a good marker for adherence junction formation in RPE cells.

**Figure 6:**
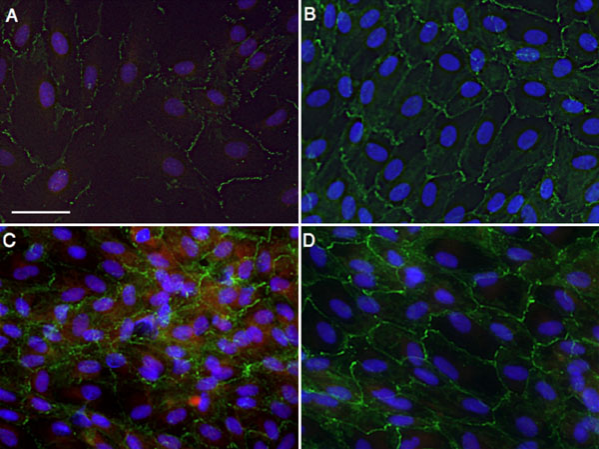
Impact of cell density and TGF-β stimulation on Snail and N-cadherin expression in ARPE-19 cells. **A**: Cellular localization of Snail (red) and N-cadherin (green) at 2 days after passage. Nuclei were counterstained with Hoechst 33258 (blue). **B**: Cellular localization of Snail (red) and N-cadherin (green) at 7 days after passage. **C**, **D**: Cellular localization of Snail (red) and N-cadherin (green) after **C** TGF-β stimulation (10 ng/ml) and **D** PBS at 7 days after passage. Scale bar=50 µm.

At 2 days after passage, ARPE-19 showed an increase in Snail expression and a disrupted N-cadherin network (Figure 6A). However, once the cells established the cell-cell contact, N-cadherin expression was markedly upregulated and Snail expression decreased (Figure 6B). Furthermore, TGF-β stimulation caused upregulation of Snail expression and disruption of N-cadherin expression after establishment of cell-cell contact (Figure 6C,D).

## Discussion

In the present study, we investigate the expression and localization of EMT-associated transcriptional factors in surgically excised CNV tissues and cultured human RPE cells. The current data indicate that RPE cells migrating into CNV tissues undergo EMT changes mediated by the transcriptional factor, Snail. To our knowledge, this is the first report of the localization of Snail in CNV tissues, a hallmark of wet AMD.

During EMT, epithelial cells lose their polarity and cellular junctions and gain mesenchymal, fibroblast-like morphology and properties [13]. The cellular process is regulated by EMT-associated transcriptional factors such as Snail, Slug, Twist, and SIP1 via suppression of genes encoding adhesion molecules [23]. It has been shown in human tissues that epithelial-derived carcinoma cells and trans-differentiated epithelial cells during tissue fibrosis, both of which have a fibroblast-like morphology, express transcriptional factors relevant to EMT [37,38]. Recently, it has been proposed that EMT can be classified into 3 subtypes, type 1–3 EMTs, based on the biologic context [39]. In brief, type 1 EMTs are associated with implantation and embryo formation. Type 2 EMTs are associated with wound healing, tissue regeneration, and organ fibrosis. The type 2 EMTs begin as part of a repair-associated event that normally generates fibroblasts and other related cells. Type 3 EMTs occur in neoplastic cells that have previously undergone genetic and epigenetic changes. In ocular tissues, the type 2 EMT had been reported to play a role in the pathogenesis of proliferative vitreoretinopathy (PVR) [40,41]. Similar to the proliferative fibrous tissues formed in PVR, CNV tissues contain fibroblasts or cellular constituents with fibroblast-like morphology. In the current study, 92% of the excised CNV tissues showed Snail localization in migrated RPE cells, whereas Slug, Twist, and SIP1 were not detected. By contrast, none of the EMT-associated transcriptional factors was found in normal retinal tissues. Therefore, the current data indicate that Snail is the predominant transcriptional factor mediating EMT (type 2 EMTs) of RPE cells and is induced under the pathological condition of CNV.

Our data show that Snail expression correlates with α-SMA, an established marker for myofibroblasts, and with the amount of tissue fibrosis in CNV tissues. Previously it was reported that choroidal fibroblasts originating from mesenchymal perivascular supporting cells contributed to tissue fibrosis in CNV [42]. In addition to the fibroblasts, the current immunofluorescence study demonstrates that flattened RPE65-positive cells with a fibroblast-like phenotype also express α-SMA with nuclear localization of Snail. CNV tissues in which RPE cells showed higher nuclear localization of Snail positively correlated with the fibrosis score. Furthermore, the in vitro study showed that cultured RPE cells strongly expressed Snail with disrupted N-cadherin formation before establishing the cell-cell contact, indicating the relevance of Snail to N-cadherin formation in RPE cells. Thus, the current data suggest that the fibroblast-like cells originating, at least in part, from RPE cells undergo type 2 EMTs via the transcriptional factor Snail and contribute to the fibrotic response in the scaring of CNV in concert with fibroblasts [42].

It has been shown that EMT is triggered by cytokine signaling, for instance, TGF-β, VEGF [43], and tumor necrosis factor (TNF)-α [44], which initiate a transcriptional dedifferentiation program correlating with morphological and functional changes [45]. Alternatively, it has also been documented that RPE are transdifferentiated to a mesenchymal phenotype by cytokines, e.g., TGF-β [41] and TNF-α [44]. In accord with previous reports using other cell lines [46,47], the current data demonstrate that Snail expression was upregulated by stimulation of TGF-β and VEGF in cultured human RPE cells. Furthermore, our immunostaining data demonstrate that TGF-β induced nuclear relocalization of Snail and disrupted N-cadherin formation in cultured RPE cells. Since cellular constituents in CNV tissues have been reported to express TGF-β and VEGF [21], the current data suggest that these inflammatory cytokines may induce Snail expression in RPE cells and play a role in the tissue fibrosis of CNV.

In summary, we showed the presence of Snail in human CNV tissues and the role of VEGF and TGF-β in the upregulation and relocalization of Snail in human RPE cells. The data indicate that Snail is a major transcriptional factor for EMT of RPE cells and is involved in the mechanism of scar formation of CNV at the late stage of AMD.
